# Reproductive health knowledge among college students in Kenya

**DOI:** 10.1186/s12889-018-5760-7

**Published:** 2018-07-24

**Authors:** Samuel Mungai Mbugua, Jane Muthoni Karonjo

**Affiliations:** School of Nursing, Mount Kenya University, Thika, 342-01000 Kenya

**Keywords:** Reproductive health, Sexually transmitted infections, Young adults, Contraception

## Abstract

**Background:**

Reproductive health knowledge is vital in the growth and development of young people and this impact greatly on their educational and personal outcome as they proceed to adulthood. There has been an increasing occurrence of sexually transmitted infections in institutions of higher learning. The study sought out the strategies used by university students to prevent unplanned pregnancy and determined their knowledge of contraception methods and sexually transmitted infections in Mount Kenya University, main campus.

**Methods:**

Stratified sampling was employed. After a signed consent was obtained, a self-administered questionnaire was used to collect data. Data were explored, analyzed and percentages used to quantify the level of knowledge. Pearson’s chi-square was used to measure associations between categorical variables and independent t-test used to measure the means and relationships of continuous variables.

**Results:**

Condom use was established as the most prevalent strategy in prevention of unplanned pregnancy at 48.5 and 46.4% in prevention of STI and HIV/AIDS. Almost two thirds (58%) of respondents reported that they were conversant with only one method of contraception, 60% had knowledge of more than two types of STIs, and 62.4% indicated that they were conversant with only hospitals as facilities providing reproductive health services.

**Conclusion:**

Young people in college require educational initiatives to sensitize them on STI, methods of contraception and positive social behaviors. There is need to improve the accessibility of reproductive health services through strengthening of services provided at campus health clinics.

## Background

There are approximately 1.7 million young people globally with 86% living in developing countries [1]. Poor reproductive health of young people is a major public health concern as they represent 26% of the population in Kenya [2]. 14% of maternal mortality and 11% of all new births globally are in females aged 15–19 years, 95% of births in adolescents occurring in developing countries [3]. Among the youth, for every one man infected with HIV, there are four women [4]. 60–80% of African women infected with the virus has been through sexual intercourse. An estimated 1.3 million adolescent girls and 780,000 adolescent boys are living with the HIV virus worldwide [5]. These negative consequences in reproductive health occur presently among young people in Sub-Saharan Africa born in social plagues of poverty, HIV/AIDS and poverty [5, 6].

Half of all new HIV infections are in young adults while one quarter to half of girls in adolescence becoming mothers before 18 years [7]. By the age of 15 years, most adolescents reach sexual maturity with potential exposure to risk of sexually transmitted diseases and unplanned pregnancy in girls [8]. In Kenya, the Kenya AIDS Indicator Survey in 2007 showed that female youth had a higher likelihood of HIV infection than their male counterparts [9]. In comparison to other regions around the world, young women in Sub-Saharan Africa rank highest in regards to risk of death due to unsafe abortions with half of all overall mortality occurring in women less than the age of 25 years [10].

The sexual behaviors of young people pose long-term and short-term concerns. Their health has a significant impact on national development. Young people contribute to high maternal mortality in Africa with up to 40% of all maternal deaths in some countries [11]. With four million unsafe abortions annually, 25% occur among young people aged 19–25 years [12]. Whereas strategies must be tailored to the developmental needs of this age group and their social contexts, effective approaches are multifaceted [13]. This requires effective interventions that will concentrate on multiple spheres of health risk aiding in promotion of sound reproductive health among young people [14]. In a study by Wamoyi et al. [15] on parent-child communication about sexual and reproductive health in rural Tanzania, parents and family were crucial factors in programs aimed at reducing risky sexual behaviors in young people. Even as parents focus on abstinence, the main goal of sexual reproductive health education should be mentorship of young people capable of employing agency in the management their own sexual reproductive health [15].

Gavin et al. noted that positive youth development programs are essential in promotion of adolescent and young people’s sexual and reproductive health [16]. Data in Kenya show that continued investment in effective prevention and treatment strategies is essential to protect adolescents’ sexual and reproductive health. Sustained resource allocation in Kenya towards effective strategies in prevention and treatment approaches is vital in safe-guarding the sexual and reproductive health of young people [17].

## Methods

### Study design and sampling

This was a cross-sectional, descriptive survey among young people attending a local university in Kenya. The study utilized stratified sampling method with strata grouped according to the schools in the university’s main campus. Participants were then randomly selected across all years of study.

### Study location and population

The study was conducted in Mount Kenya University, the largest private institution of higher learning in Kenya. The university has a population of approximately 40,000 students in its main campus, with students coming from various catchment areas, where the study was conducted. There were 8 schools found within the main campus from which the 338 participants were sampled. Table 1 outlines the sample representation from each school based on student populations in each.

**Table 1:** Sample representation from each school

School	Student population	Frequency	% of Sample population
Nursing	550	5	1.5
Medicine	540	5	1.5
Education	10,550	89	26.3
Social Sciences	5797	49	14.5
Pure and Applied Sciences	7150	61	18.0
Business and Economics	9637	81	24.0
Pharmacy	532	5	1.5
Health Sciences	4997	43	12.7
Total	39,753	338	100.0

### Data collection and analysis

Data were collected by a team of trained nurses using a self-administered questionnaire with instructions on how to respond. A formal standardized questionnaire design was used in line with the research objectives, the demographic data preceding the objectives. Both open-ended and closed-ended questions were posed depending on the variable. Pre-testing was carried out, involving 10% of the sample population (35 participants), in Gretsa university which has a similar student population characteristic as Mount Kenya university. The questionnaire sought to assess the knowledge on unplanned pregnancy, sexually transmitted infections preventive strategies, contraceptive methods, negative behavior factors in reproductive health and facilities where they can seek reproductive health services. The key outcome variable was establishing knowledge of reproductive health among university students. Statistical analysis was done using Statistical Package for Social Sciences (SPSS) version 20. Percentages were used to establish the reproductive health knowledge among college students. Pearson’s chi-square was used to measure associations between categorical variables and independent t-test used to measure the means and relationships of continuous variables.

## Results

All 338 participants completed the questionnaires with males constituting 50.3 and 49.7% representing females. Out of the total, 20.1% of the respondents were aged 20 years, with a similar finding in those aged 21, 16.6% aged 23 years, 16.3% aged 22 years, 13.3% aged 24 years, and 8.6% aged 19 years while 5.0% aged 18 years. Majority of the respondents (35.2%) were in their third year of study, 30.5% in first year, 21.3% in second year and 13.0% in their fourth year of study.

### Knowledge of strategies likely employed in prevention of unplanned pregnancy

**Fig. 1:**
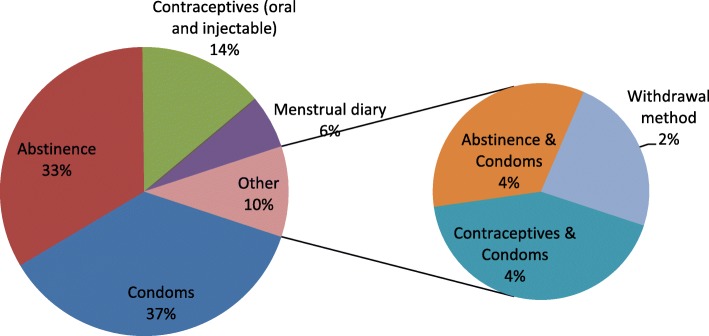
Strategies in Prevention of STIs

Abstinence from sexual practices was recognized by 33% of the respondents. Use of condoms as a strategy in prevention of unplanned pregnancy was the most prevalent at 37% of sexually active respondents. Approximately 14% of the sexually active students identified oral and injectable contraceptives, 2% reported the likely use of withdrawal method. This is represented in Fig. 1.

### Knowledge of strategies likely employed in prevention of sexually transmitted infections (STI)

**Fig. 2:**
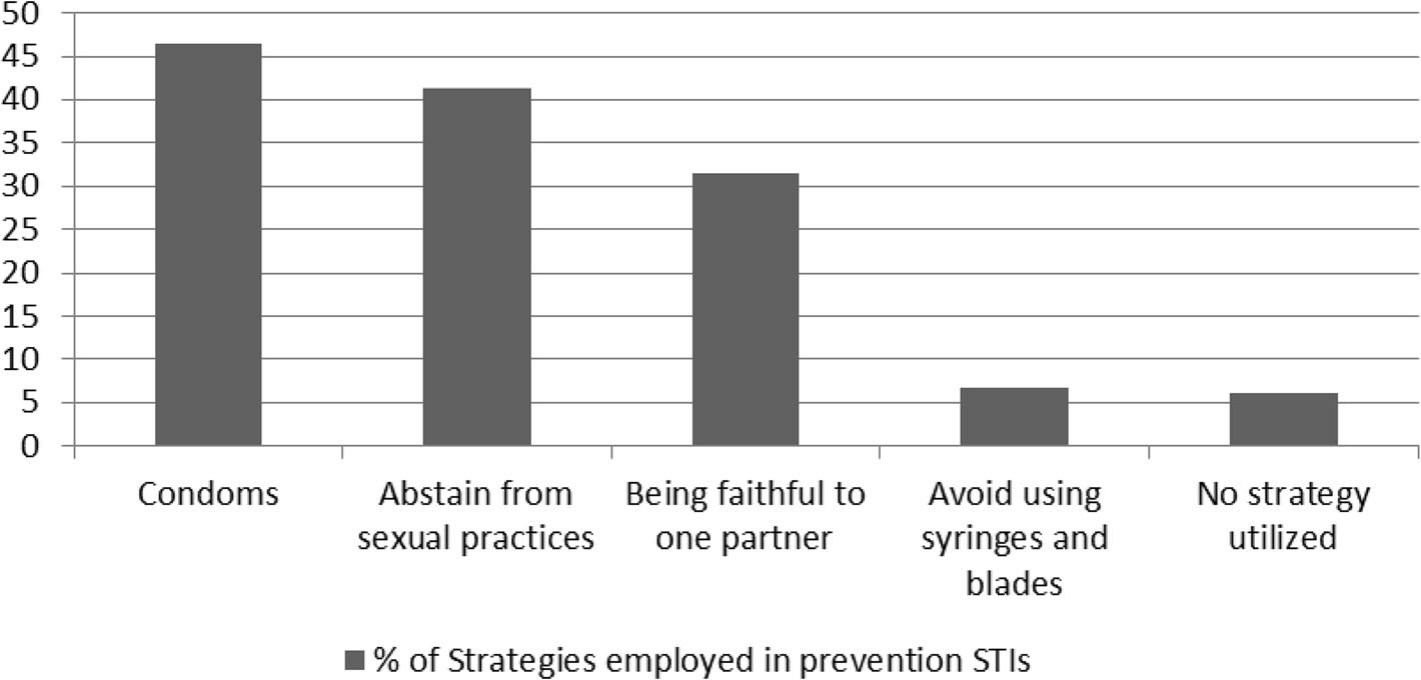
Strategies employed to prevent unplanned pregnancy

Use of condoms as a strategy in prevention of HIV and other STI was the most prevalent strategy reported in 46.4% of the respondents followed by abstinence from sexual practices (41.4%). Faithfulness to one partner was reported at 31.2%. This is illustrated Fig. 2.

### Knowledge of contraception methods

**Fig. 3:**
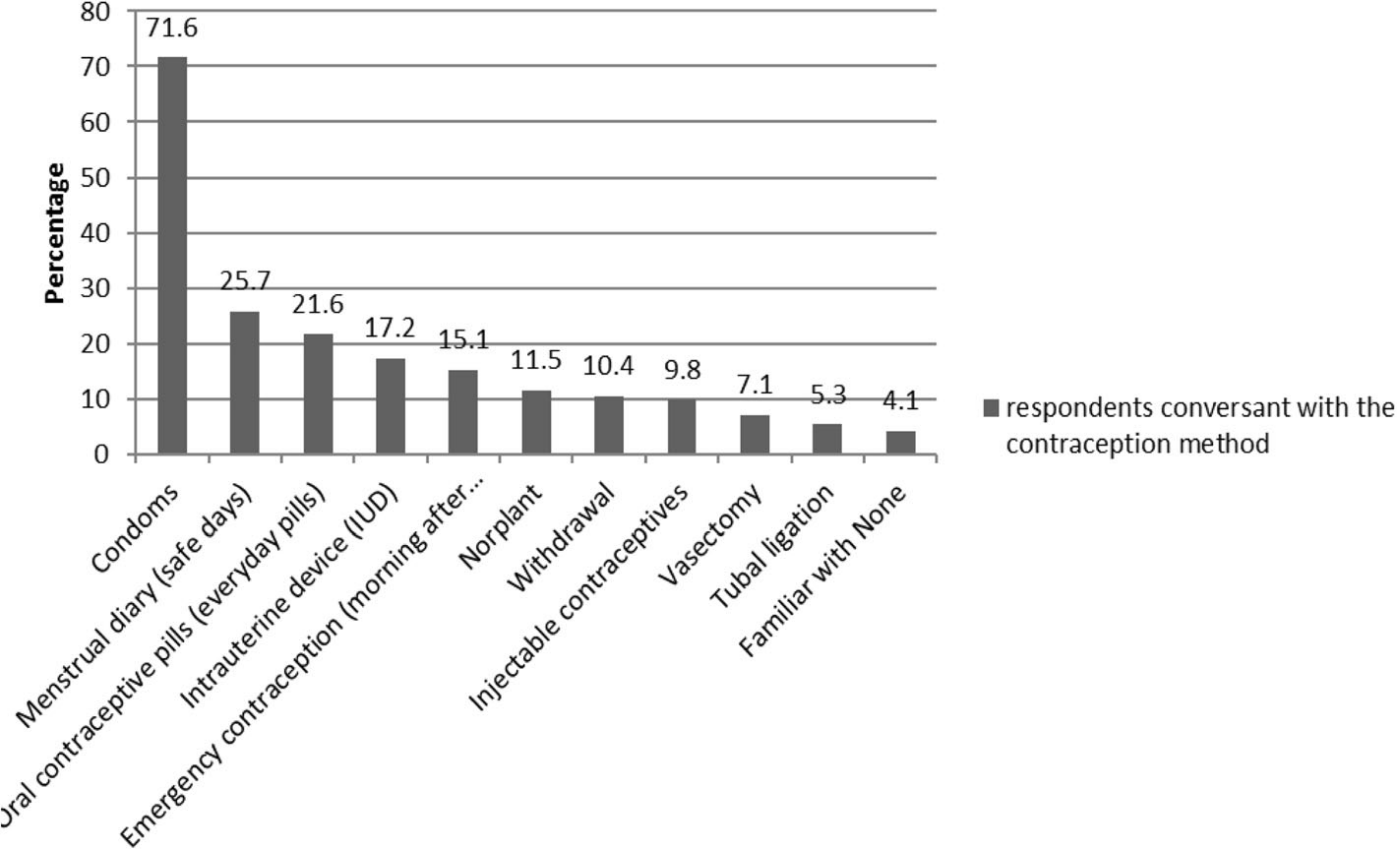
Respondents conversant with each contraception method

When asked which method of contraception they were conversant with, 71.6% of the respondents stated that they had adequate knowledge about use of condoms, 25.7% asserted they were conversant with the menstrual diary while 9.8% were conversant with injectable contraceptives. 7.1% were conversant with vasectomy and tubal ligation was only known to 5.3% of the respondents. 4.1% of the respondents were not conversant with any method of contraception despite extensive campaigns. (Fig. 3).

It was established that 82.1% of the female respondents were aware of their HIV status compared to 76.5% of their male counterparts. Out of the total, 60% of respondents were conversant with two or more sexually transmitted infections while 39.4% were aware of only one sexually transmitted infection. From the 60% that could identify two or more sexually transmitted infections, approximately 41.4% were conversant with HIV/AIDS, gonorrhea, syphilis, genital warts & herpes, close to 25% were conversant with only HIV/AIDS, gonorrhea and syphilis while 9.8% with HIV/AIDS, gonorrhea, syphilis and herpes (Table 2).

**Table 2:** Knowledge of more than one Sexually Transmitted Infection (60% of respondents, *n* = 203)

STI (s)- More than one	Frequency	Percent
HIV/AIDS, Gonorrhea, Syphilis, Genital warts & Herpes	84	41.4
HIV/AIDS, Gonorrhea & Syphilis	50	24.7
HIV/AIDS, Gonorrhea, Syphilis & Herpes	20	9.8
Gonorrhea & Syphilis	10	4.9
Gonorrhea, Syphilis, Genital warts & Herpes	10	4.9
HIV/AIDS, Gonorrhea, Syphilis & Genital warts	7	3.5
HIV/AIDS & Syphilis	5	2.5
HIV/AIDS & Gonorrhea	5	2.5
Gonorrhea, Syphilis & Genital warts	3	1.5
Gonorrhea, Syphilis & Herpes	3	1.5
HIV/AIDS & Herpes	2	0.9
HIV/AIDS & Genital warts	2	0.9
HIV/AIDS, Syphilis & Herpes	1	0.5
Genital warts & Herpes	1	0.5

Participants were also queried about their knowledge of behavioral factors negatively affecting the reproductive health of the youth attending Mount Kenya University. The representation of responses is shown in Table 3 with 41.7% citing drug and substance abuse as the main trait.

**Table 3:** Knowledge of individual or combined behavioral factors negatively affecting reproductive health

Factor (n = 338)	Frequency	Percent
Drug and substance abuse	141	41.7
Risky sexual practices (orgies, homosexuality, multiple sex partners, and casual sex)	66	19.5
Sexual violence (e.g. rape)	43	12.7
Combination of all the above	88	26

### Knowledge on facilities providing reproductive health services

**Fig. 4:**
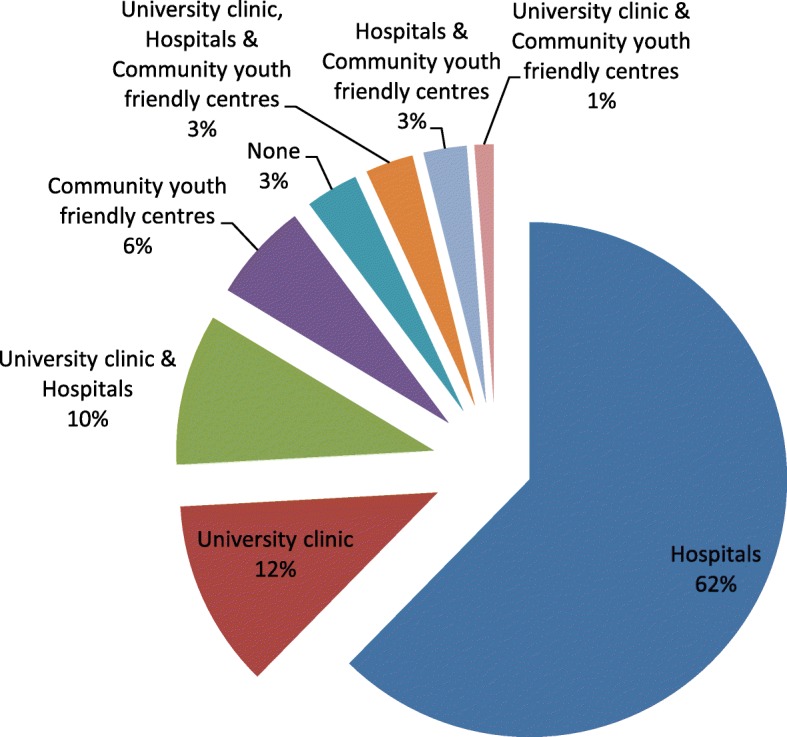
Knowledge of facilities where students could access reproductive health services

It was established that 62.4% were conversant with hospitals only, 11.8% were aware of the university clinic only, while 9.5% were conversant with both the hospitals and the university clinic as facilities providing reproductive health (Fig. 4).

Chi square test was performed to measure the association between sexual experiences and gender. Significance was established with females exhibiting a higher likelihood of responsible sexual experiences than their male counterparts, χ = 10.62, df = 3, *p* = .014. When asked if they had any sexual experience, 84.1% of males reported of having experience with sex compared to 71.4% of females. 59.5% of the female respondents sought reproductive health services from hospitals as did 54.1% of males. It was further established that 25.9 and 25.6% of the males and females respectively sought out services in reproductive health from the University clinic.

## Discussion

Understanding the knowledge on reproductive health is an important facet in the lives of college students with a direct implication on completion of university studies and progression to fecund adults. The findings indicated a deficiency in knowledge regarding reproductive health in college students meaning that there was inadequate knowledge on various reproductive health matters such as STIs, contraception methods and facilities providing reproductive health.

Condom use and sexual abstinence were the main strategies recognized in prevention of unplanned pregnancy and STI. This correlates with Pascual et al. [18] and Ayehu et al. [19] reporting abstinence as the main preventive strategy. Pascual et al. [18] established the findings from conducting 55 systematic reviews while Ayehu et al. [19] used a cross sectional study using a sample size of 781 in the Awabel District of Ethiopia through multi stage sampling. Condom use was also the most prevalent HIV/AIDS preventive method among the students consistent with the study by Protogerou et al. [20] on the integrated theory of condom use for young people in Sub-Saharan Africa. Majority of respondents reported one or two contraceptive methods as having adequate knowledge about condoms as the method they were most conversant; with Selassie [21] reporting similar findings in Ethiopia. In regards to sexually transmitted infections, data indicates most students possessed knowledge on more than one STI with a slightly higher percentage of females reporting of awareness of their HIV status compared to their male counterparts. This correlates to Grulich et al. [22] Drug and substance abuse was cited as the most prevalent negative behavior affecting reproductive health, correlating with Asante et al. in Ghana in a study on substance abuse and risky sexual behavior among youth in Accra [23]. Knowledge of facilities offering reproductive health services showed that hospitals were the most recognized source of reproductive health services followed by the university clinic. This is similar to Thatte et al. [24] in a study on barriers to reproductive health services in Ghana, Dickson-Tetteh et al. [25] in South Africa and Godia et al. [26] in Kenya.

Female students depicted a likelihood of responsible sexual experience compared to the males, similar to Denno et al. [27]. Most young people sought reproductive health services from hospitals and the university clinics. This correlates to studies by Woog et al. [28] on adolescent women’s need for and use of sexual and reproductive health services in developing countries. We acknowledge the limitations of this study which was a quantitative study in a private university in Kenya and the findings cannot be generalized to all institutions of higher education in Kenya. Experiences and learning points are all the same shared across institutions and countries. This study relied on data from a self-administered questionnaire which is prone to bias especially in regards to sexual health.

## Conclusion

This study showed that young people in university showed inadequate comprehension in reproductive health knowledge with variations in knowledge on preventive strategies of STI and unplanned pregnancy. Directional educational initiatives are vital in sensitization of university students and young people on contraception methods, prevention of sexually transmitted infections and constructive social behaviors and norms during their university life. It is crucial to improve the accessibility of reproductive health services to young people with establishment of a youth friendly community center within the learning institution being a fundamental requirement. From these findings, investigative studies using qualitative methods on sexual and reproductive health communication between young people in university and their parents/guardians should be conducted.

## Abbreviations

HIV: Human immunodeficiency virus
HIV/AIDS: Human immunodeficiency virus/ Acquired immunodeficiency syndrome
SPSS: Statistical Package for Social Sciences
STI: Sexually transmitted Infections
Young Adults: Young adults are defined as persons aged 18–25 years
